# Short-term responses across two sequential plant-based activity sessions: an exploratory fixed-order pilot study

**DOI:** 10.3389/fpsyg.2026.1962154

**Published:** 2026-10-02

**Authors:** Li-li Chang, Yun-Hsien Ku, Chuen-Yu Chuang, Yu-Sen Chang

**Affiliations:** Department of Horticulture and Landscape Architecture, National Taiwan University, Taipei, Taiwan

**Keywords:** DASS-21, electroencephalography, equivalence testing, heart-rate variability, horticultural activity, nature-based intervention, pilot study, state anxiety

## Abstract

**Background:**

Structured plant-based activities combine natural materials, purposeful manual work, sensory engagement, and social interaction, but direct comparisons of practical formats in community adults remain limited. We characterized short-term responses during two sequential sessions and compared within-session changes.

**Methods:**

Twenty-five adults participated in a single-group, fixed-order pilot study. Session 1 involved horticultural crafting; 1 week later, Session 2 involved balm crafting followed by horticultural crafting. Twenty-three attended both sessions. DASS-21 and STAI-S were principal outcomes; horticultural wellbeing, HRV, and forehead single-channel EEG were exploratory. Within-session changes and complete-case between-session differences were estimated with 95% CIs. *Post hoc* TOST examined exploratory equivalence bounds.

**Results:**

Both sessions were followed by favorable short-term psychological changes. DASS-21 scores decreased by 8.57 points in Session 1 [95% CI (5.41, 11.72), *n* = 23] and 5.53 in Session 2 [(2.65, 8.41), *n* = 17]. STAI-S scores decreased by 7.30 points in Session 1 [(5.23, 9.37), *n* = 25] and 6.86 in Session 2 [(4.07, 9.66), *n* = 22]; all four Holm-adjusted *p* values were <0.001. Complete-case comparisons showed no statistically detectable between-session difference in DASS-21 improvement [3.12 points, 95% CI (−1.51, 7.75)] or STAI-S improvement [0.34 points, (−3.15, 3.83)]. STAI-S changes met the exploratory TOST criterion only at the broad ±0.50 standardized bound. Horticultural wellbeing improved in both sessions; SDNN and RMSSD increased, whereas EEG findings did not remain significant after multiplicity correction.

**Conclusion:**

Both sessions were associated with favorable short-term psychological changes, particularly reduced distress and state anxiety. These findings support the feasibility and potential value of brief, hands-on plant-based activities in community settings and provide estimates for larger randomized, counterbalanced trials. Given the fixed order and absence of a control group, the findings are preliminary rather than definitive causal or modality-specific evidence.

## 1 Introduction

Accessible plant-based group activities are increasingly used in community, educational, and wellness settings. Gardening and horticultural-therapy studies have reported improvements in depression, anxiety, stress, and broader health outcomes (Soga et al., 2017; Lu et al., 2023; Wood et al., 2025). Plant-based activities may be attractive because they combine contact with natural materials, structured manual work, social interaction, and completion of a tangible product. These components are also consistent with Stress Recovery Theory and Attention Restoration Theory, which describe restorative responses to contact with natural environments (Ulrich et al., 1991; Kaplan, 1995).

Horticultural intervention is not a single, uniform exposure. A broad review catalogued outdoor gardening, indoor planting, crafts made with live or pressed plant materials, flower arranging, and other plant-related activities; most programs combined more than one activity and varied in session number and length (Park et al., 2016). Later syntheses likewise coded setting, activity type, frequency, session duration, and total course length (Lu et al., 2023; Chen et al., 2025). These reviews identify delivery characteristics as plausible sources of heterogeneity, but subgroup contrasts across heterogeneous studies should not be interpreted as causal head-to-head evidence. In community-dwelling adults, for example, meta-analytic subgroup estimates were broadly similar for indoor and outdoor programs and for once- versus twice-weekly delivery, amid diverse intervention components and substantial risk-of-bias concerns (Sin et al., 2025). A recent small comparative study of four- and eight-session programs further showed why immediate and cumulative outcomes should be distinguished (Liu et al., 2026).

Some primary studies have directly varied specific features of horticultural delivery. Son et al. (2004) contrasted field gardening, indoor container culture, and indoor flower design among adults with chronic schizophrenia. Han (2018) compared active care of classroom plants with passive exposure among junior-high students; active care produced greater self-reported stress restoration, whereas both groups reported attention restoration. Short experiments in young adults have compared multiple tasks and cognitive-demand levels (Kim and Park, 2023), and Guo et al. (2024) compared five hands-on activities in children, finding broadly similar physiological recovery alongside some activity-specific affective patterns. Comparative work therefore exists, but it is dispersed across clinical, child, and laboratory samples and often manipulates one feature or a brief task. Evidence directly comparing fully specified, multicomponent hands-on packages in community adults, while separating activity content from sequence, duration, facilitator contact, expectancy, and setting, remains limited.

The present community pilot included two sessions delivered 1 week apart. The first session was a horticultural potted-arrangement activity. The second combined balm crafting with a subsequent horticultural activity. This contrast does not isolate aroma: the second session added a different manual task, new materials, an additional product, and a fixed sequence. Furthermore, every participant received the sessions in the same order. Activity package was therefore inseparable from visit order, familiarity, day-to-day variation, and possible carryover.

We consequently reframed the study as descriptive and hypothesis-generating. The first objective was to estimate short-term pre–post changes within each session, emphasizing the validated DASS-21 and STAI-S. The second was to describe whether complete-case change scores differed between sessions and how precise those differences were. The third was to examine, strictly exploratorily, whether the observed change differences fell within standardized TOST bounds and whether conclusions changed under narrower bounds. The fourth was to summarize phase-specific Session 2 results and exploratory HRV and EEG patterns. No confirmatory modality-equivalence or causal hypothesis was tested.

## 2 Materials and methods

### 2.1 Design and setting

This was a single-group, fixed-order, repeated-measures pilot. All participants attended Session 1 on October 20, 2024; Session 2 occurred 1 week later on October 27, 2024. There was no untreated, wait-list, attention, or active control condition, and participants and facilitators were not masked. The fixed sequence is shown in Figure 1. Because the activity package and session order were perfectly confounded, between-session analyses describe observed patterns but cannot identify a modality effect.

**Figure 1:**
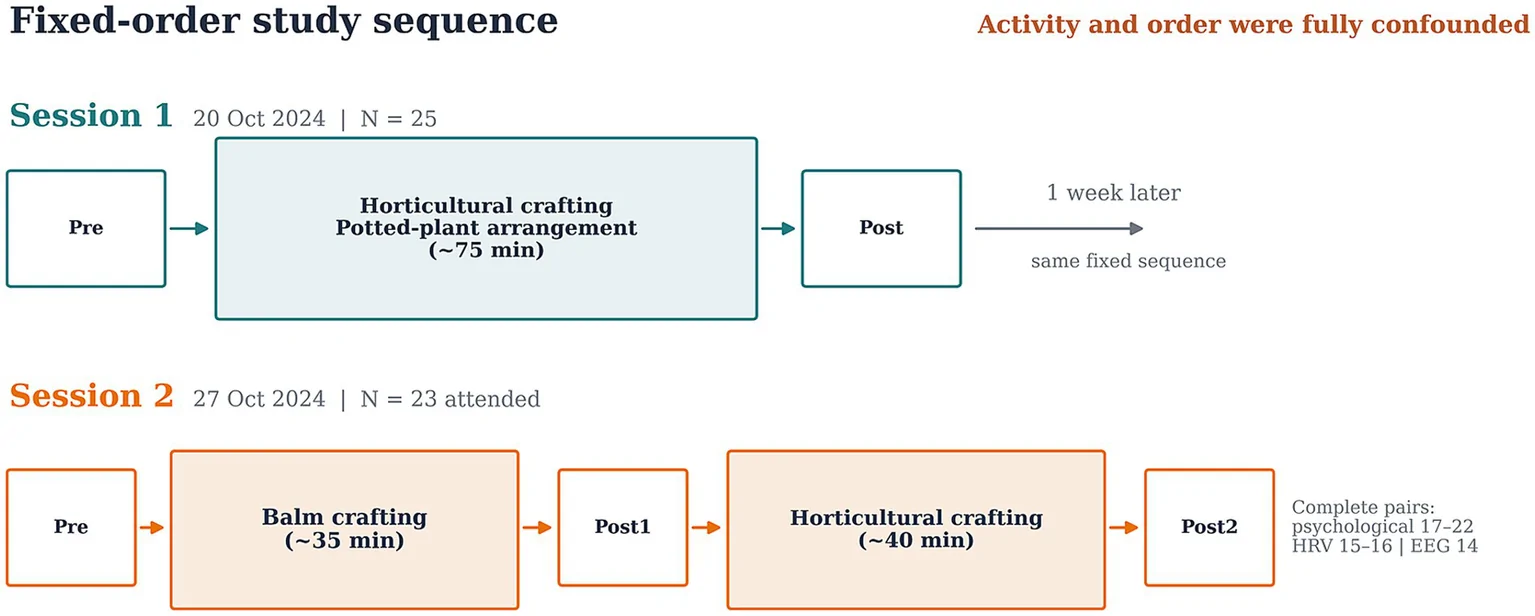
Fixed-order study design and analyzable psychological samples. Session 2 always followed Session 1 by 1 week; the design therefore cannot separate activity package from order, familiarity, carryover, or day-to-day effects.

### 2.2 Participants

Participants were recruited from community colleges and adult-education programs. Eligibility criteria were age ≥20 years, ability to communicate in Chinese and complete questionnaires, stable health, nonpregnant/nonlactating status, and no known previous adverse reaction to the horticultural materials, tea, or essential oils; known cognitive impairment preventing participation was an exclusion criterion. Twenty-five adults enrolled (6 men and 19 women; mean age 46.9 ± 11.9 years, range 23–74), and 23 attended Session 2. Seventeen of 25 participants (68.0%) reported an agriculture or horticulture background at Session 1; among Session 2 attendees, 16 of 23 (69.6%) reported such a background. The Session 1 and Session 2 samples are not independent groups (Table 1).

**Table 1:** Participant characteristics and analysis availability.

Characteristic	Enrolled/Session 1	Attended Session 2
Participants	25	23
Sex, men/women	6/19	6/17
Age, mean ± SD (range), years	46.9 ± 11.9 (23–74)	47.3 ± 12.0 (23–74)
Agriculture / horticulture background	17/25 (68.0%)	16/23 (69.6%)
Horticultural wellbeing complete pre–post	23	22
DASS-21 complete pre–post	23	17
STAI-S complete pre–post	25	22
HRV complete pre–post	24	16
EEG complete pre–post	19	14

Session 2 values describe the subset of the Session 1 cohort who returned; columns should not be interpreted as independent groups. Effective sample size varied because of session nonattendance, questionnaire incompleteness, or inadequate physiological signal quality. No missing values were imputed.

### 2.3 Activity sessions

Both sessions lasted approximately 75 min, were conducted as group activities, and were led by the same facilitation team. Session 1 comprised an introduction to plant materials (approximately 10 min), a demonstration (10 min), hands-on potted-arrangement work using foliage plants (40 min), and sharing of completed work (15 min). Questionnaires and five-minute seated physiological recordings were obtained before and after the session.

Session 2 comprised two sequential phases: balm crafting (approximately 35 min) and potted-plant arrangement (approximately 40 min). The balm was prepared as a common batch using 700 mL distilled water; 75 mL mixed carrier oils, reported by the activity author as olive, grapeseed, shea-derived, and rosehip oils; 20 g menthol dissolved in the carrier oils in advance; wintergreen, lavender, tea tree, and eucalyptus essential oils (4, 4, 3, and 2 mL, respectively); and 10 mL of a poly-γ-glutamic-acid-derived emulsifier. The final total essential-oil concentration was approximately 1.6%. All participants used the same formulation and concentration; the exact individual aliquot volume was not retained. Under facilitator demonstration, all participants applied the balm to the skin and used it during massage. Materials were explained and prior allergies or adverse reactions were checked before participation. A mid-session assessment (Post1) followed balm crafting, and a final assessment (Post2) followed horticultural crafting. The same team instructed and supervised both sessions, although facilitator contact and other activity components were not experimentally matched. No verbatim standardized instruction script was archived, and no adverse event was reported.

### 2.4 Outcomes

#### 2.4.1 Principal descriptive psychological outcomes

The 21-item Depression Anxiety Stress Scales (DASS-21) were administered in Chinese (Lovibond and Lovibond, 1995; Moussa et al., 2017). The doubled total score (range 0–126) was analyzed; lower scores indicate less distress. State anxiety was assessed with the 20-item State–Trait Anxiety Inventory–State form (STAI-S; Spielberger et al., 1983; range 20–80), using the Chinese version described by Shek (1993); lower scores indicate less state anxiety. The DASS-21 and STAI-S were treated as the principal descriptive outcomes in this revision. This hierarchy was not preregistered and does not convert the study into a confirmatory trial.

#### 2.4.2 Exploratory horticultural wellbeing questionnaire

A seven-item Chinese program-evaluation questionnaire adapted from Kuo and Chen (2012) assessed perceived physical vigor, interest, relaxation, accomplishment, decision-making, social skills, and horticultural cultivation. Items were rated from 1 (strongly disagree) to 5 (strongly agree); no explicit retrospective recall period was printed on the instrument. A 7–35 total was calculated only when all seven items were present, with higher scores indicating more favorable perceived wellbeing. The total was retained as an exploratory program-evaluation summary, not a validated unidimensional scale. Time-specific coefficient-alpha estimates ranged from 0.49 to 0.87 in complete-item samples of 22–24 and are too unstable to establish reliability or validity. Complete literal English translations of the administered Chinese items, scoring, distributions, and ceiling observations are provided in Supplementary Tables S1, S2.

#### 2.4.3 Exploratory physiological outcomes

HRV was recorded with a BeneGear EB-810 analyzer (BeneGear Inc., Taiwan). Five-minute seated segments yielded SDNN, RMSSD, LF/HF, and normalized high-frequency power (nHF). EEG was recorded for 5 min with a BeneGear forehead-worn single-channel device; the active and reference contacts were positioned approximately at Fp1 and Fp2, respectively. The archived software export contained device-derived values labeled delta, theta, alpha-low, alpha-mid, alpha-high, beta-low, beta-mid, beta-high, gamma, Thinking, and Concentration at approximately 6.27-s intervals. Archived post-processing documentation indicates that time points associated with movement values exceeding 200 μV were flagged and all band values at the corresponding time points were excluded. A complete auditable rejection log and the manufacturer’s signal-processing documentation were not retained; consequently, the rejected-epoch proportion, filter settings, spectral-estimation procedure, epoch overlap, signal units, and numerical sub-band cutoffs cannot be reported reliably. Thinking and Concentration were generated automatically by proprietary software and were treated only as exploratory device-derived indices. HRV conditions were not documented sufficiently to control respiration, recent intake and activity, medication, time of day, cardiac ectopy, or room conditions. The physiological analyses cannot identify a specific autonomic or neural mechanism.

### 2.5 Statistical analysis

Analyses were reproduced from the authoritative participant-level master workbook using Python 3.12.13, pandas 2.2.3, NumPy 2.3.5, and SciPy 1.17.0. Positive change was defined as post minus pre for horticultural wellbeing and pre minus post for DASS-21 and STAI-S, so that positive values consistently denote improvement. Horticultural wellbeing totals required all seven items; unreadable or missing item responses were not scored as zero. Analyses used available paired observations for each specified contrast, reported the exact n, and did not impute missing data.

Within-session psychological changes were summarized as pre and post means ± SD, raw mean improvement with 95% CI, paired t test, and paired-samples Cohen’s *d*_z_ with an approximate small-sample 95% CI (Cohen, 1988). The four principal descriptive tests (DASS-21 and STAI-S in two sessions) formed one Holm family. Horticultural wellbeing, item-level, phase-specific, DASS-21 subscale, HRV, and EEG analyses were exploratory. Individual five-point wellbeing items were summarized descriptively. For HRV and EEG, Holm adjustments were applied separately within each session and outcome family; log1p-transformed paired tests and Wilcoxon signed-rank sensitivity analyses were examined for skewed distributions.

For participants with complete pre and post data in both sessions, we compared the Session 1 and Session 2 improvement scores using paired t tests and reported raw mean differences, 95% CIs, and *d*_z_ Baselines were compared descriptively. As a missing-data diagnostic, Session 1 baseline scores were compared between complete and incomplete two-session cases using Welch tests; these small comparisons have low power and do not establish missing-at-random assumptions. As a sensitivity analysis, each outcome was stacked as up to four observations per participant (Session 1 pre and post; Session 2 pre and final post) and estimated by ordinary least squares with participant indicators, session, time, and session × time. The interaction described the difference in pre–post changes. Participant-clustered covariance used the conventional CR1 finite-sample multiplier [G/(G − 1)] × [(N − 1)/(N − K)], and inference used a Student t reference distribution with G − 1 degrees of freedom, where G is the number of participant clusters, N the number of observations, and K the number of estimated coefficients. No additional CR2 or wild-cluster correction was applied. No model can remove the structural confounding between session and activity.

Equivalence analyses were exploratory and *post hoc* (Schuirmann, 1987; Lakens et al., 2018). TOST was applied to complete-case Session 1 minus Session 2 change differences. Because no validated minimally important raw-score differences were available, the standardized bound *d*_z_ = ±0.50 was treated as an illustrative pilot criterion rather than a clinically or operationally justified margin. For each outcome, that standardized bound was converted to raw units using the observed SD of paired change differences and was therefore data-dependent. We report both one-sided t statistics and *p* values, 90% CIs, raw bounds, and sensitivity analyses using *d*_z_ = ±0.30 and ±0.20. Nonsignificant difference tests were not interpreted as evidence of equivalence.

Normality of paired differences was examined with Shapiro–Wilk tests and outliers with the 1.5 × IQR rule. Given the small sample, distribution diagnostics and sensitivity tests are interpreted descriptively. Three-time-point Session 2 total-score analyses were restricted to complete observations and examined with Friedman tests and paired Wilcoxon contrasts; these analyses were exploratory and the contrasts were not preregistered. For each outcome separately, the three contrasts (Pre vs. Post1, Post1 vs. Post2, and Pre vs. Post2) constituted one three-test Holm family; no adjustment was made across the three outcomes or combined with other exploratory families. All tests were two-sided at α = 0.05 except the two one-sided equivalence tests, each evaluated at *α* = 0.05. The study was not preregistered, and no prospective equivalence sample-size calculation was performed. For orientation only, we recalculated the minimum complete-pair sample sizes using exact finite-sample power for an unknown-variance one-sample t-based TOST applied to paired differences. Under normally distributed paired differences, a true standardized difference of *d*_z_ = 0, symmetric bounds, *α* = 0.05 for each one-sided test, and joint TOST power of at least 0.80, the smallest n values were 36 for *d*_z_ = ±0.50 (power = 0.805), 97 for *d*_z_ = ±0.30 (0.803), and 216 for *d*_z_ = ±0.20 (0.801). Power was evaluated by exact integration over the independent standard-normal sampling distribution of the mean and chi-square sampling distribution of the variance; recruitment would need to be larger to allow for attrition.

### 2.6 Ethics

The National Taiwan University Research Ethics Committee for Behavioral and Social Sciences approved the study (NTU-REC No. 202304HM014). All participants provided written informed consent.

## 3 Results

### 3.1 Attendance, missing data, and baselines

All 25 enrolled participants attended Session 1, and 23 attended Session 2. Questionnaire completeness differed by instrument: 23 participants had all seven horticultural wellbeing items at both Session 1 assessments, 22 provided complete horticultural wellbeing and STAI-S data in Session 2, and 17 provided complete DASS-21 data in Session 2. Two Session 1 horticultural wellbeing records contained unreadable item responses and were excluded from total-score analyses rather than treating those items as zero. The records did not document why other individual questionnaires were incomplete; respondent burden cannot be assumed as the cause. Physiological complete pairs were smaller because of signal quality (Table 1). Baseline comparisons and complete-versus-incomplete diagnostics appear in Supplementary Tables S3, S4.

Among complete two-session cases, Session 1 minus Session 2 baseline differences were −0.45 points for horticultural wellbeing [95% CI (−1.69, 0.79), *p* = 0.456], 3.88 for DASS-21 (−0.61, 8.38), *p* = 0.086, and 1.25 for STAI-S (−2.02, 4.52), *p* = 0.436. These imprecise estimates show that starting levels were not identical, especially for distress, and support analysis of within-session changes rather than endpoints alone.

### 3.2 Within-session psychological changes

DASS-21 and STAI-S scores decreased from pre to post in both sessions (Table 2; Figure 2). In Session 1, mean DASS-21 improvement was 8.57 points [95% CI (5.41, 11.72); **d*_z_* = 1.18], and mean STAI-S improvement was 7.30 [5.23, 9.37] (**d*_z_* = 1.46). In Session 2, mean improvements were 5.53 [2.65, 8.41] for DASS-21 (**d*_z_* = 0.99) and 6.86 [4.07, 9.66] for STAI-S (**d*_z_* = 1.09). All four Holm-adjusted *p* values were <0.001. These within-session associations cannot be separated from passage of time, repeated testing, social interaction, expectancy, or other uncontrolled influences.

**Table 2:** Within-session changes in psychological outcomes.

Outcome	Session	*n*	Pre, *M* ± SD	Post, *M* ± SD	Improvement [95% CI]	t (df)	*p*	Holm *p*	*d*_z_ [95% CI]
DASS-21	Session 1	23	16.74 ± 11.63	8.17 ± 8.00	8.57 [5.41, 11.72]	5.64 (22)	<0.001	<0.001	1.18 [0.61, 1.74]
DASS-21	Session 2	17	12.47 ± 12.43	6.94 ± 12.06	5.53 [2.65, 8.41]	4.07 (16)	<0.001	<0.001	0.99 [0.35, 1.62]
STAI-S	Session 1	25	38.14 ± 7.63	30.84 ± 8.23	7.30 [5.23, 9.37]	7.28 (24)	<0.001	<0.001	1.46 [0.86, 2.05]
STAI-S	Session 2	22	36.36 ± 8.30	29.50 ± 10.20	6.86 [4.07, 9.66]	5.11 (21)	<0.001	<0.001	1.09 [0.52, 1.65]
Horticultural wellbeing	Session 1	23	23.17 ± 3.19	26.22 ± 3.73	3.04 [1.76, 4.33]	4.90 (22)	<0.001	—	1.02 [0.48, 1.56]
Horticultural wellbeing	Session 2	22	24.05 ± 2.59	27.59 ± 4.59	3.55 [2.08, 5.01]	5.03 (21)	<0.001	—	1.07 [0.51, 1.63]

Positive values denote improvement. DASS-21 and STAI-S are the principal descriptive outcomes; their four session-specific tests form one Holm family. Horticultural wellbeing is exploratory and is not included in that family. Positive improvement equals pre − post for DASS-21/STAI-S and post − pre for wellbeing. *d*_z_ = paired-samples Cohen’s d.

**Figure 2:**
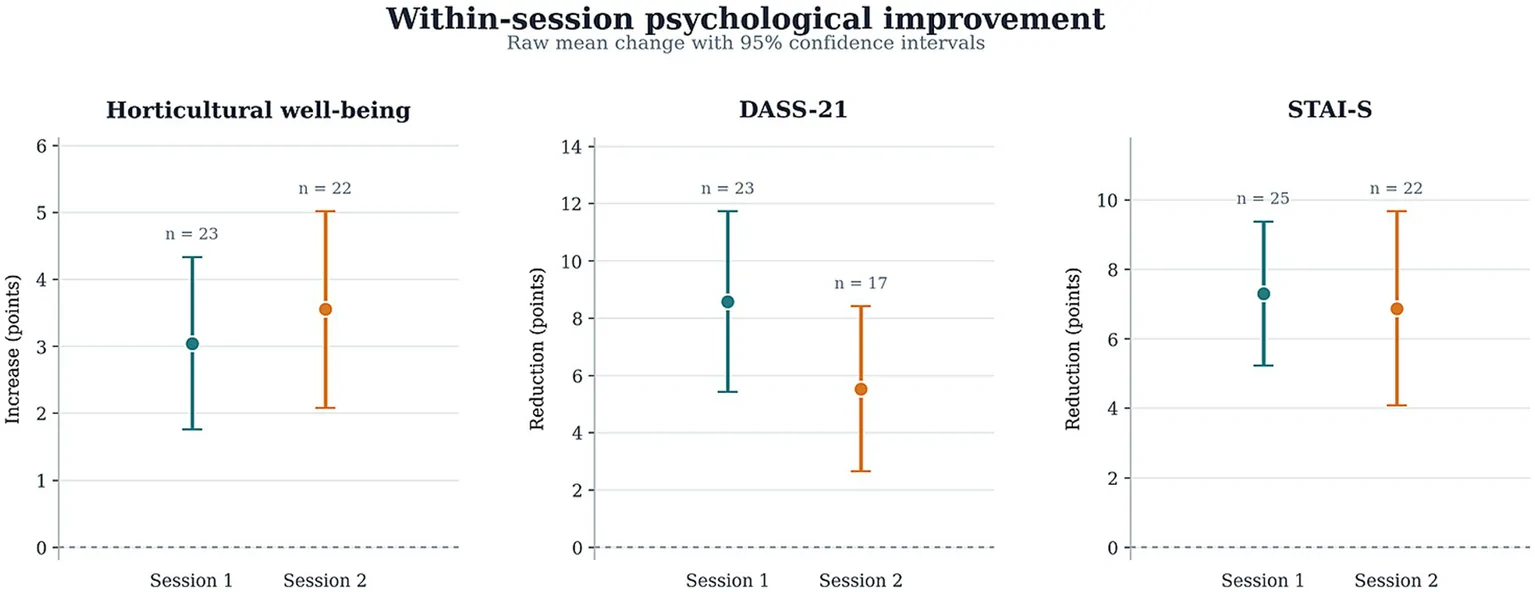
Raw mean within-session improvement with 95% confidence intervals. Positive values denote improvement. Horticultural wellbeing is exploratory; DASS-21 and STAI-S are principal descriptive outcomes.

Exploratory horticultural wellbeing scores increased by 3.04 points in Session 1 [95% CI (1.76, 4.33); *n* = 23] and 3.55 points in Session 2 (2.08, 5.01); *n* = 22. The corresponding Wilcoxon sensitivity tests were also consistent with pre–post shifts (both *p* < 0.001). Because the questionnaire is not independently validated, these estimates describe this program-evaluation measure only.

At the item level, the largest Session 1 mean increases were in accomplishment (+0.80), relaxation (+0.71), and horticultural cultivation (+0.64), whereas interest/hobbies was unchanged. From Session 2 Pre to Post2, accomplishment and logical thinking/decision-making increased most (both +0.77), followed by relaxation (+0.59), horticultural cultivation (+0.45), and social skills (+0.41); interest/hobbies changed least (+0.23). These ordinal item patterns are descriptive and cannot identify a mechanism (Supplementary Table S8A).

### 3.3 Complete-case comparison of session changes

The complete-case change differences were small relative to their uncertainty for horticultural wellbeing and STAI-S and larger but imprecise for DASS-21 (Table 3). Session 1 minus Session 2 improvement was −0.60 points for wellbeing [95% CI (−2.90, 1.70); *n* = 20], 3.12 for DASS-21 (−1.51, 7.75); *n* = 17, and 0.34 for STAI-S (−3.15, 3.83); *n* = 22. All CIs crossed zero. These results neither demonstrate a session difference nor establish similarity; the intervals define the range of differences compatible with the small complete-case samples.

**Table 3:** Complete-case comparison of Session 1 and Session 2 improvement scores.

Outcome	*n*	Session 1 improvement, *M* ± SD	Session 2 improvement, *M* ± SD	S1 − S2 [95% CI]	*t* (df)	*p*	*d*_z_ [95% CI]
Horticultural wellbeing	20	3.05 ± 3.14	3.65 ± 3.36	−0.60 [−2.90, 1.70]	−0.54 (19)	0.592	−0.12 [−0.58, 0.34]
DASS-21	17	8.65 ± 6.44	5.53 ± 5.60	3.12 [−1.51, 7.75]	1.43 (16)	0.173	0.35 [−0.18, 0.88]
STAI-S	22	7.20 ± 5.33	6.86 ± 6.30	0.34 [−3.15, 3.83]	0.20 (21)	0.841	0.04 [−0.40, 0.49]

Analyses include only participants with complete pre and post data in both sessions. A nonsignificant difference test is not evidence of equivalence. Fixed order prevents attribution of any similarity or difference to activity package.

### 3.4 Exploratory equivalence and sensitivity analyses

At the illustrative *d*_z_ = ±0.50 bound, the exploratory TOST criterion was met only for STAI-S; it was not met for horticultural wellbeing or DASS-21 (Table 4; Figure 3). The raw bounds were ±2.46 wellbeing points, ±4.50 DASS-21 points, and ±3.94 STAI-S points because each was calculated from the observed SD of paired change differences. For wellbeing, the lower one-sided test narrowly missed the criterion (*p* = 0.054); for DASS-21, the upper one-sided test was nonsignificant (*p* = 0.267). The STAI-S result was not robust to narrower margins: none of the three outcomes met both one-sided criteria at *d*_z_ = ±0.30 or ±0.20. The results therefore do not support a general claim of equivalent activity effects.

**Table 4:** Exploratory TOST of complete-case change differences using the illustrative *d*_z_ = ±0.50 bound.

Outcome	*n*	Raw bound	S1 − S2 [90% CI]	*t* lower (df)	*p* lower	t upper (df)	*p* upper	Result
Horticultural wellbeing	20	±2.46	−0.60 [−2.50, 1.30]	1.69 (19)	0.054	−2.78 (19)	0.006	Not met
DASS-21	17	±4.50	3.12 [−0.70, 6.93]	3.49 (16)	0.002	−0.63 (16)	0.267	Not met
STAI-S	22	±3.94	0.34 [−2.55, 3.23]	2.55 (21)	0.009	−2.14 (21)	0.022	Criterion met

The ±0.50 standardized bound was selected *post hoc* and is not a validated minimally important difference. The raw bound equals 0.50 × the observed SD of the paired Session 1 minus Session 2 change difference. Both one-sided p values must be <0.05 for the criterion to be met. Meeting this statistical criterion does not resolve fixed-order confounding or establish practical interchangeability.

**Figure 3:**
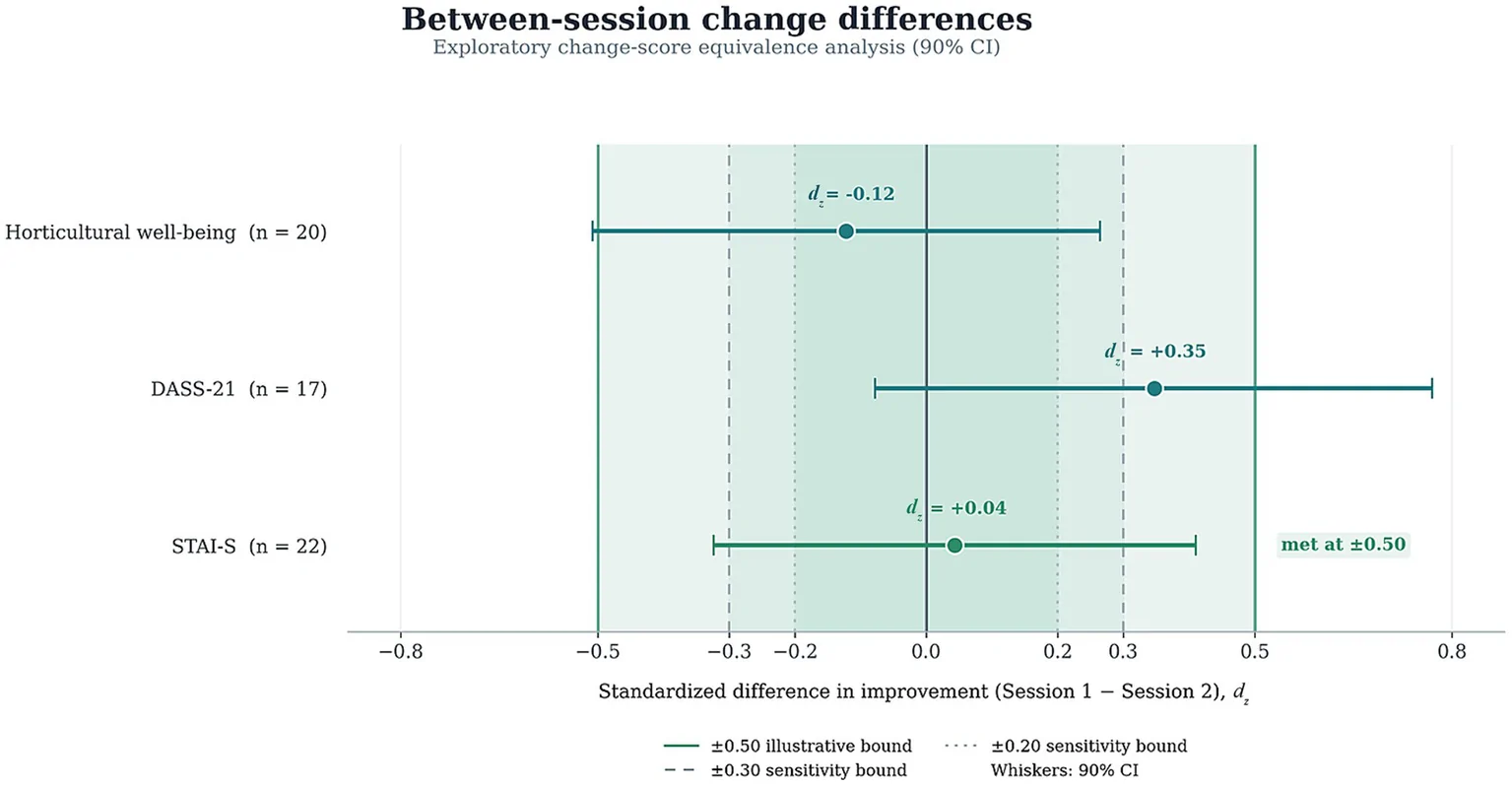
Complete-case Session 1 minus Session 2 mean change differences and 90% confidence intervals. Shading denotes the exploratory *d*_z_ = ±0.50 criterion after data-dependent conversion to raw units; dashed and dotted lines show the narrower ±0.30 and ±0.20 sensitivity bounds. No outcome met the TOST criterion at either narrower bound.

### 3.5 Repeated-observation and missing-data sensitivity analyses

Participant fixed-effects sensitivity models retained all available pre and post observations. The horticultural wellbeing model included 91 observations from 25 participants (*K* = 28; cluster df = 24), the DASS-21 model included 80 observations from 23 participants (*K* = 26; cluster df = 22), and the STAI-S model included 94 observations from 25 participants (*K* = 28; cluster df = 24). Estimated Session 1 minus Session 2 improvement differences were −0.45 points for horticultural wellbeing [95% CI (−2.89, 1.98), *p* = 0.703], 3.04 for DASS-21 [95% CI (−2.27, 8.34), *p* = 0.248], and 0.44 for STAI-S [95% CI (−3.48, 4.35), *p* = 0.820]. These estimates were consistent with the complete-case comparisons but remained imprecise. Session 1 baseline comparisons between complete and incomplete two-session cases were nonsignificant for wellbeing (*p* = 0.485), DASS-21 (*p* = 0.830), and STAI-S (*p* = 0.371); the small incomplete groups limit this diagnostic.

### 3.6 Phase-specific and physiological observations

Within Session 2, exploratory Friedman tests indicated overall differences across Pre, Post1, and Post2 for horticultural wellbeing (*χ*^2^ = 22.24, *n* = 22, *p* < 0.001), DASS-21 (*χ*^2^ = 18.63, *n* = 17, *p* < 0.001), and STAI-S (*χ*^2^ = 23.49, *n* = 22, *p* < 0.001). The larger numerical changes occurred from Pre to Post1, following balm crafting: mean improvements were 2.64 points for horticultural wellbeing (Holm *p* = 0.002), 4.47 points for DASS-21 (Holm *p* = 0.005), and 5.32 points for STAI-S (Holm *p* = 0.001). Additional improvements from Post1 to Post2, following the subsequent horticultural activity, were smaller: 0.91 points for horticultural well-being (Holm *p* = 0.341), 1.06 points for DASS-21 (Holm *p* = 0.201), and 1.55 points for STAI-S (Holm *p* = 0.116). These phase-specific analyses were exploratory and were not preregistered (Supplementary Table S7).

For HRV, SDNN increased in Session 1 by 18.40 ms [95% CI (12.50, 24.30), Holm *p* < 0.001] and in Session 2 by 13.54 ms (5.89, 21.18), Holm *p* = 0.007. RMSSD increased by 5.50 ms [95% CI: 1.35, 9.65] in Session 1 (Holm *p* = 0.035) and by 9.07 ms [3.84, 14.30] in Session 2 (Holm *p* = 0.007). RMSSD change distributions were skewed, but log-transformed and Wilcoxon sensitivity analyses were directionally consistent. LF/HF and nHF did not change after correction. These short, incompletely standardized recordings are compatible with altered HRV during the sessions but cannot establish improved autonomic regulation or a mechanism.

In Session 1, several EEG outcomes had nominal *p* values <0.05, including beta-low power, but no EEG outcome remained significant after Holm correction; log1p-transformed and Wilcoxon sensitivity analyses also provided no corrected evidence. Session 2 EEG comparisons were nonsignificant. Large dispersion, small samples, single-channel acquisition, incomplete processing documentation, and proprietary indices preclude specific neural, attentional, or flow interpretations. Full exploratory results are in Supplementary Tables S10, S11.

## 4 Discussion

### 4.1 Principal findings

In this fixed sequence, DASS-21 and STAI-S scores improved from pre to post in both sessions. Complete-case change differences were not statistically distinguishable from zero, but their CIs were wide, particularly for DASS-21. Exploratory TOST results depended on both outcome and bound: the ±0.50 criterion was met only for STAI-S, and no outcome met the criterion under narrower bounds. This pattern is evidence of uncertainty, not evidence that the two activity packages are interchangeable.

The revised interpretation differs fundamentally from an endpoint-equivalence claim. Endpoints answer whether participants finished at similar observed levels; change scores ask whether their pre–post shifts were similar. In the present data, the Session 2 DASS-21 baseline was numerically lower than the Session 1 baseline among complete cases. Similar endpoints could therefore coexist with different changes. Reporting both baselines and change estimates makes this distinction visible, although neither approach removes order confounding.

### 4.2 What the fixed sequence can and cannot show

Every participant completed the horticultural-crafting session first and the combined activity session second. Any similarity or difference between sessions may reflect familiarity with the research setting, changed expectations, reduced questionnaire reactivity, carryover, regression to the mean, day-to-day variation, or selective completion. These are structural design limitations that cannot be corrected by a different statistical model.

Within Session 2, the larger numerical changes in horticultural wellbeing, distress, and state anxiety occurred during the first phase, after balm crafting, whereas the additional changes following the subsequent horticultural activity were smaller. This exploratory pattern should not be interpreted as evidence that the second activity was ineffective. Balm crafting always preceded horticultural crafting, and participants had already reported favorable changes before beginning the second phase. The pattern could therefore reflect differences in activity characteristics, position within the fixed sequence, repeated exposure to the setting or assessments, reduced room for further improvement after the initial response, or other temporal dynamics. The present design cannot distinguish among these explanations or attribute the early change specifically to balm making, aroma exposure, or any isolated component. Future studies should randomize or counterbalance the within-session phase order—and preferably the session-package order—to separate activity-specific effects from sequence-related influences.

The sessions also differed as complex packages. Session 2 added manual balm preparation, unfamiliar materials, olfactory exposure, an extra tangible product, and a two-phase sequence. The study therefore does not isolate essential-oil exposure or compare shared manual engagement with a single content-specific mechanism. A mechanistic design would independently manipulate aroma and manual activity, match duration and facilitator contact, use an olfactory control, and randomize or counterbalance both session and phase order.

### 4.3 Statistical and practical interpretation

Equivalence margins should represent the largest difference that stakeholders consider unimportant. We did not have a validated minimally important difference, prospective expert consensus, or operational threshold for any outcome. A conventional standardized value of 0.50 is therefore an illustrative design sensitivity only. In paired data, its raw-score equivalent depends on the SD of within-person change differences; using the observed SD makes the margin sample-dependent. The failure of every outcome at ±0.30 and ±0.20 demonstrates fragility and argues against a definitive equivalence statement.

The raw CIs are more useful for planning. For DASS-21, the complete-case data remain compatible with Session 1 improvement being about 1.5 points smaller to 7.8 points larger than Session 2 improvement. For STAI-S, the compatible range is approximately 3.1 points smaller to 3.8 points larger. Stakeholders should define acceptable raw-score differences before a future trial. Under the exact finite-sample paired-TOST calculation described in Methods, the illustrative standardized ±0.50 bound and a true zero difference require 36 complete pairs—not 17–22—for at least 80% power; attrition and narrower margins would increase recruitment requirements.

### 4.4 Psychological and physiological context

The direction of the within-session psychological changes is consistent with broader reports of short-term stress and anxiety improvement after horticultural or creative nature-based activities (Soga et al., 2017; Lu et al., 2023; Chung et al., 2024; Wood et al., 2025). However, without any control condition, the present study cannot attribute those changes to the activities. Social interaction, attention from facilitators, expectancy, repeated measurement, and simple passage of time remain viable explanations.

SDNN and RMSSD increased in both sessions, but physiological mechanism claims require caution. Five-minute HRV can be influenced by breathing, posture, time of day, recent intake and activity, medication, and signal editing. Several relevant controls were not documented. EEG results were still more uncertain: none survived family-wise correction, and the device was single-lead with incompletely archived processing. We therefore removed interpretations linking beta power to flow or claiming improved autonomic regulation. The physiological findings are descriptive signals for better controlled research, not confirmation of psychological effects.

### 4.5 Measurement and missingness

The horticultural wellbeing questionnaire was useful for program evaluation but is not a validated co-primary outcome. Its seven items span distinct perceived domains; the time-specific alpha estimates varied from 0.49 to 0.87 in very small samples and neither prove unidimensionality nor establish validity. The summed score is therefore reported exploratorily only when all seven items were present, alongside full item wording and distributions. Total-score ceiling rates were low, but the sample is too small for stable psychometric conclusions.

Missingness was outcome-specific and most pronounced for Session 2 DASS-21, with only 17 complete pairs. The reason was not documented, so we removed speculation that questionnaire burden caused noncompletion. Baseline comparisons between complete and incomplete cases did not detect differences, but these tests were underpowered and cannot rule out informative missingness. No imputation was performed; exact sample sizes and complete-case definitions are reported throughout.

### 4.6 Strengths, limitations, and next study

Strengths include repeated measurement of the same cohort, reporting of exact analyzable samples, joint psychological and physiological observation, reconstruction from participant-level data, effect estimates with CIs, explicit change-score analysis, and equivalence sensitivity checks. The pilot also identified practical reporting needs for a larger study.

Limitations are substantial: fixed and uncounterbalanced order; absence of untreated, attention, or active controls; nonmasked participation; small and predominantly female community sample; high prior horticultural exposure; two brief sessions; unmeasured expectations and social-context effects; outcome-specific missingness; a nonvalidated exploratory questionnaire; no archived verbatim activity script or exact individual balm dose; incompletely standardized HRV; and single-channel EEG with limited processing reproducibility. The outcome hierarchy and equivalence analyses were specified during revision rather than preregistered.

A definitive study should randomize a larger sample to counterbalanced sequences; prospectively designate one or a small number of validated outcomes; define raw-score equivalence margins using stakeholder and clinical judgment; record expectations and adverse events; match duration, novelty, tangible products, and facilitator contact; and use an appropriate control. A factorial design could separate manual activity from aroma exposure. Physiological acquisition should standardize respiration, posture, intake, activity, medication, time, and environment and archive transparent signal-processing criteria.

## 5 Conclusion

In a small fixed-order pilot, participants reported short-term psychological changes after both a horticultural-crafting session and, 1 week later, a combined balm-crafting and horticultural-crafting session. Complete-case estimates did not detect clear differences between the two sessions, but uncertainty was wide, and the single exploratory TOST result at the broadest bound disappeared under narrower bounds. Because session order was completely confounded with activity package and no control group was included, the data do not establish causal benefit, modality equivalence, or an aroma-specific effect. The appropriate contribution of this study is a transparent set of descriptive estimates and methodological lessons for a randomized, counterbalanced investigation.

## Data Availability

The anonymized data supporting the findings of this study, together with a data dictionary and analysis code, are available from the corresponding author upon reasonable request, subject to the conditions of participant consent and ethics approval.
