# Supplementary material for: Short-term responses across two sequential plant-based activity sessions: an exploratory fixed-order pilot study
Source: Front Psychol. 2026 Oct 2;17:1962154. doi: 10.3389/fpsyg.2026.1962154 (PMC13672660; doi:10.3389/fpsyg.2026.1962154)
Supplement: Supplementary file 1 [file Data_Sheet_1.PDF]

Supplementary Material

Supplementary Tables S1–S11 provide the questionnaire wording and scoring information, additional psychological analyses, fixed-effects and equivalence sensitivity analyses, and complete exploratory HRV and EEG results referenced in the article. No participant names or direct identifiers are included in this supplementary report.

Table S1. Seven-item horticultural well-being program-evaluation questionnaire.

Domain	Literal English translation of administered Chinese item	Response scale
Physical vigor	My physical movement is sufficiently strong.	1 strongly disagree; 2 disagree; 3 neutral/fair; 4 agree; 5 strongly agree
Interest/hobbies	I have interests and hobbies.	1 strongly disagree; 2 disagree; 3 neutral/fair; 4 agree; 5 strongly agree
Relaxation	I feel relaxed.	1 strongly disagree; 2 disagree; 3 neutral/fair; 4 agree; 5 strongly agree
Accomplishment	I have a sense of accomplishment.	1 strongly disagree; 2 disagree; 3 neutral/fair; 4 agree; 5 strongly agree
Logical thinking/decision-making	My logical thinking and judgment are strong, and I can readily make correct decisions.	1 strongly disagree; 2 disagree; 3 neutral/fair; 4 agree; 5 strongly agree
Social skills	I have good social skills.	1 strongly disagree; 2 disagree; 3 neutral/fair; 4 agree; 5 strongly agree
Horticultural cultivation	I am skilled in horticultural cultivation techniques.	1 strongly disagree; 2 disagree; 3 neutral/fair; 4 agree; 5 strongly agree

Note. The questionnaire was administered in Chinese and adapted from Kuo and Chen (2012); the table provides literal English translations for reproducibility. No explicit retrospective recall period was printed on the instrument. The seven same-direction items were summed only when all were present (range 7–35); the total is an exploratory program summary, not a validated unidimensional scale.

Table S2. Horticultural well-being total-score distributions, ceiling observations, and time-specific internal consistency.

Assessment	n	Mean ± SD	Median [IQR]	Range	At maximum 35	Coefficient α
Session 1 Pre	23	23.17 ± 3.19	23.0 [22.0, 25.0]	14–28	0/23 (0.0%)	0.57 (n=23)
Session 1 Post	24	26.50 ± 3.90	26.5 [25.0, 28.2]	15–33	0/24 (0.0%)	0.78 (n=24)
Session 2 Pre	22	24.05 ± 2.59	23.0 [22.0, 26.5]	21–28	0/22 (0.0%)	0.49 (n=22)
Session 2 Post1	22	26.68 ± 3.75	26.0 [24.0, 28.0]	21–35	1/22 (4.5%)	0.82 (n=22)
Session 2 Post2	22	27.59 ± 4.59	28.5 [24.0, 31.0]	21–35	1/22 (4.5%)	0.87 (n=22)

Note. Alpha estimates vary markedly across small time-specific complete-item samples and do not establish construct validity or unidimensionality.

Table S3. Baseline comparison among participants with complete pre–post data in both sessions.

Outcome	n	Session 1 Pre	Session 2 Pre	S1 − S2 [95% CI]	t (df)	p
Horticultural well-being	20	23.55 ± 2.65	24.00 ± 2.47	-0.45 [-1.69, 0.79]	-0.76 (19)	.456
DASS-21	17	16.35 ± 10.77	12.47 ± 12.43	3.88 [-0.61, 8.38]	1.83 (16)	.086
STAI-S	22	37.61 ± 7.75	36.36 ± 8.30	1.25 [-2.02, 4.52]	0.79 (21)	.436

Note. Baseline testing is descriptive. A nonsignificant baseline test does not establish equality; the fixed order remains structurally confounded.

Table S4. Session 1 baseline comparison between complete and incomplete two-session cases.

Outcome	Complete n	Complete, M ± SD	Incomplete n	Incomplete, M ± SD	Welch t (df)	p
Horticultural well-being	20	23.55 ± 2.65	3	20.67 ± 5.86	0.84 (2.12)	.485
DASS-21	17	16.35 ± 10.77	6	17.83 ± 14.91	-0.22 (6.94)	.830
STAI-S	22	37.61 ± 7.75	3	42.00 ± 6.56	-1.06 (2.83)	.371

Note. The incomplete groups are extremely small, and these tests cannot establish missing-at-random assumptions. For horticultural well-being, participants lacking a complete seven-item Session 1 baseline are not included in this diagnostic.

Table S5. Sensitivity of exploratory change-score TOST to standardized equivalence bounds.

Outcome	n	dz bound	Raw bound	S1 − S2 [90% CI]	p lower	p upper	Criterion
Horticultural well-being	20	±0.50	±2.46	-0.60 [-2.50, 1.30]	.054	.006	Not met
Horticultural well-being	20	±0.30	±1.48	-0.60 [-2.50, 1.30]	.218	.037	Not met
Horticultural well-being	20	±0.20	±0.98	-0.60 [-2.50, 1.30]	.365	.083	Not met
DASS-21	17	±0.50	±4.50	3.12 [-0.70, 6.93]	.002	.267	Not met
DASS-21	17	±0.30	±2.70	3.12 [-0.70, 6.93]	.008	.574	Not met
DASS-21	17	±0.20	±1.80	3.12 [-0.70, 6.93]	.019	.722	Not met
STAI-S	22	±0.50	±3.94	0.34 [-2.55, 3.23]	.009	.022	Met
STAI-S	22	±0.30	±2.36	0.34 [-2.55, 3.23]	.061	.121	Not met
STAI-S	22	±0.20	±1.57	0.34 [-2.55, 3.23]	.133	.235	Not met

Note. Bounds were post hoc and were converted with the observed SD of paired change differences. Both one-sided p values must be <.05. No outcome met the criterion at ±0.30 or ±0.20.

Table S6. All-available participant fixed-effects sensitivity estimates.

Outcome	Participants	Observations	S1 − S2 improvement [95% CI]	p
Horticultural well-being	25	91	-0.45 [-2.89, 1.98]	.703
DASS-21	23	80	3.04 [-2.27, 8.34]	.248
STAI-S	25	94	0.44 [-3.48, 4.35]	.820

Note. Each ordinary least-squares model included participant indicators, session, time, and session × time. Inference used participant-clustered CR1 covariance with the finite-sample multiplier $[G/(G − 1)] × [(N − 1)/(N − K)]$ and a Student t reference distribution with $G − 1$ degrees of freedom; no additional CR2 or wild-cluster correction was applied. Here G, N, and K denote participant clusters, observations, and estimated coefficients, respectively: horticultural well-being, $G = 25$, $N = 91$, $K = 28$; DASS-21, $G = 23$, $N = 80$, $K = 26$; and STAI-S, $G = 25$, $N = 94$, $K = 28$. These estimates use all available observations but cannot remove activity-by-order confounding.

Table S7. Exploratory three-time-point Session 2 total-score analyses.

Outcome	n	Pre, M ± SD	Post1, M ± SD	Post2, M ± SD	Friedman χ^2	p
Horticultural well-being	22	24.05 ± 2.59	26.68 ± 3.75	27.59 ± 4.59	22.24	<.001
DASS-21	17	12.47 ± 12.43	8.00 ± 11.58	6.94 ± 12.06	18.63	<.001
STAI-S	22	36.36 ± 8.30	31.05 ± 8.65	29.50 ± 10.20	23.49	<.001

Note. Friedman tests are exploratory. Post1 always followed balm crafting and Post2 always followed the subsequent horticultural activity.

Outcome	n	Contrast	Mean improvement	Wilcoxon W	Nominal p	Holm p
Horticultural well-being	22	Pre → Post1	2.64	14.5	.001	.002
Horticultural well-being	22	Post1 → Post2	0.91	56.5	.341	.341
Horticultural well-being	22	Pre → Post2	3.55	3.5	<.001	<.001
DASS-21	17	Pre → Post1	4.47	4.0	.002	.005
DASS-21	17	Post1 → Post2	1.06	15.0	.201	.201
DASS-21	17	Pre → Post2	5.53	0.0	.001	.004
STAI-S	22	Pre → Post1	5.32	9.5	<.001	.001
STAI-S	22	Post1 → Post2	1.55	49.5	.116	.116
STAI-S	22	Pre → Post2	6.86	10.5	<.001	.001

Note. Positive mean values denote improvement. For each outcome separately, the Pre vs. Post1, Post1 vs. Post2, and Pre vs. Post2 contrasts constituted one three-test Holm family; no adjustment was made across outcomes or combined with other exploratory families. Fixed phase order prevents attribution to activity content.

Table S8A. Exploratory horticultural well-being item patterns.

Item	S1 n	S1 Pre	S1 Post	S1 Δ	S2 n	S2 Pre	S2 Post1	S2 Post2	Pre→P1 Δ	P1→P2 Δ	Pre→P2 Δ
Physical vigor	25	3.12 ± 0.83	3.32 ± 1.03	0.20	22	3.18 ± 0.80	3.45 ± 1.01	3.50 ± 0.91	0.27	0.05	0.32
Interest/hobbies	24	4.00 ± 0.72	4.00 ± 0.66	0.00	22	4.05 ± 0.79	4.23 ± 0.61	4.27 ± 0.77	0.18	0.05	0.23
Relaxation	24	3.58 ± 0.83	4.29 ± 0.62	0.71	22	3.82 ± 0.66	4.41 ± 0.59	4.41 ± 0.80	0.59	0.00	0.59
Accomplishment	25	3.48 ± 0.71	4.28 ± 0.61	0.80	22	3.59 ± 0.73	4.14 ± 0.56	4.36 ± 0.79	0.55	0.23	0.77
Logical thinking/decision-	25	3.32 ± 0.99	3.68 ± 1.03	0.36	22	3.32 ± 0.78	3.73 ± 0.70	4.09 ± 0.81	0.41	0.36	0.77

Item	S1 n	S1 Pre	S1 Post	S1 Δ	S2 n	S2 Pre	S2 Post1	S2 Post2	Pre→P1 Δ	P1→P2 Δ	Pre→P2 Δ
making											
Social skills	25	3.04 ± 0.89	3.40 ± 0.82	0.36	22	3.09 ± 0.75	3.45 ± 0.91	3.50 ± 0.96	0.36	0.05	0.41
Horticultural cultivation	25	2.72 ± 0.84	3.36 ± 0.95	0.64	22	3.00 ± 0.69	3.27 ± 0.88	3.45 ± 1.01	0.27	0.18	0.45

Note. Items are ordinal five-point responses and are summarized descriptively. Positive changes indicate a more favorable rating. No mechanistic interpretation is assigned to item patterns.

Table S8B. Exploratory DASS-21 subscale changes.

Subscale	Session	n	Pre, M ± SD	Post, M ± SD	Improvement [95% CI]	Nominal p	dz
Depression	Session 1	23	3.74 ± 4.31	1.57 ± 2.43	2.17 [0.89, 3.46]	.002	0.73
Depression	Session 2	17	3.24 ± 4.28	1.71 ± 4.12	1.53 [0.57, 2.49]	.004	0.82
Anxiety	Session 1	23	5.30 ± 4.08	3.26 ± 3.08	2.04 [0.96, 3.12]	<.001	0.82
Anxiety	Session 2	17	3.88 ± 4.47	2.24 ± 3.65	1.65 [0.44, 2.85]	.010	0.70
Stress	Session 1	23	7.70 ± 4.36	3.35 ± 3.24	4.35 [2.75, 5.94]	<.001	1.18
Stress	Session 2	17	5.35 ± 4.51	3.00 ± 4.60	2.35 [0.88, 3.83]	.004	0.82

Note. Subscales are exploratory; nominal p values are reported for transparency and are not part of the four-test principal Holm family.

Table S9. Exploratory HRV pre–post results and distribution-sensitive checks.

Session	Outcome	n	Pre, M ± SD	Post, M ± SD	Post − Pre [95% CI]	Paired t p	Holm p	log1p p	Wilcoxon p
Session 1	SDNN (ms)	24	40.15 ± 15.18	58.55 ± 24.04	18.40 [12.50, 24.30]	<.001	<.001	<.001	<.001
Session 1	RMSSD (ms)	24	24.06 ± 14.11	29.56 ± 21.33	5.50 [1.35, 9.65]	.012	.035	.003	.007
Session 1	LF/HF (ratio)	24	3.20 ± 1.81	2.96 ± 1.38	-0.24 [-0.92, 0.44]	.473	.946	.623	.565
Session 1	nHF (%)	24	29.38 ± 11.31	30.08 ± 12.45	0.70 [-4.33, 5.72]	.776	.946	.754	.922
Session 2	SDNN (ms)	16	37.87 ± 18.73	51.41 ± 23.92	13.54 [5.89, 21.18]	.002	.007	.002	.002
Session 2	RMSSD (ms)	16	20.79 ± 15.53	29.86 ± 21.91	9.07 [3.84, 14.30]	.002	.007	<.001	<.001
Session 2	LF/HF (ratio)	16	3.55 ± 1.77	2.93 ± 1.53	-0.62 [-1.36, 0.13]	.097	.193	.082	.105
Session 2	nHF (%)	16	26.78 ± 11.43	30.44 ± 13.43	3.66 [-0.83, 8.14]	.103	.193	.126	.117

Note. Holm adjustment was applied to the four raw-scale HRV tests within each session. Log1p and Wilcoxon p values are sensitivity checks. Short recordings and incompletely documented covariates preclude a specific autonomic mechanism.

Table S10. Exploratory EEG raw-scale pre–post results.

Session	Device-derived output	n	Pre, M ± SD	Post, M ± SD	Post − Pre [95% CI]	Nominal p	Holm p	dz
Session 1	Delta	19	1469.34 ± 1968.26	2420.90 ± 3589.18	951.56 [47.67, 1855.45]	.040	.308	0.51
Session 1	Theta	19	124.73 ± 242.89	171.44 ± 277.52	46.71 [-29.29, 122.71]	.213	.910	0.30
Session 1	Alpha-low	19	14.29 ± 26.61	18.99 ± 25.44	4.71 [-2.42, 11.83]	.182	.910	0.32
Session 1	Alpha-mid	19	27.24 ± 28.42	41.20 ± 48.44	13.96 [1.33, 26.59]	.032	.289	0.53
Session 1	Alpha-high	19	17.45 ± 24.01	22.81 ± 25.26	5.36 [0.12, 10.60]	.045	.308	0.49
Session 1	Beta-low	19	24.22 ± 24.99	33.33 ± 32.27	9.11 [2.12, 16.10]	.014	.149	0.63
Session 1	Beta-mid	19	25.95 ± 27.09	29.44 ± 29.57	3.49 [-3.63, 10.62]	.317	.910	0.24
Session 1	Beta-high	19	53.16 ± 58.80	52.24 ± 62.04	-0.93 [-17.89, 16.04]	.910	.910	-0.03
Session 1	Gamma	19	411.58 ± 312.25	497.53 ± 567.47	85.95 [-76.49, 248.38]	.281	.910	0.26
Session 1	Thinking index	19	54.86 ± 47.17	80.19 ± 78.17	25.33 [3.04, 47.62]	.028	.282	0.55
Session 1	Concentration index	19	667.07 ± 794.93	1101.99 ± 1413.62	434.92 [25.63, 844.21]	.039	.308	0.51
Session 2	Delta	14	2928.69 ± 3337.07	2899.92 ± 5297.28	-28.77 [-2031.39, 1973.86]	.976	1.000	-0.01
Session 2	Theta	14	314.06 ± 514.03	461.70 ± 1053.35	147.64 [-403.85, 699.13]	.573	1.000	0.15
Session 2	Alpha-low	14	30.01 ± 52.33	41.20 ± 91.13	11.20 [-35.29, 57.69]	.612	1.000	0.14
Session 2	Alpha-mid	14	65.06 ± 108.51	82.90 ± 149.86	17.84 [-57.85, 93.53]	.619	1.000	0.14
Session 2	Alpha-high	14	26.78 ± 31.70	36.68 ± 70.05	9.89 [-27.02, 46.81]	.572	1.000	0.15
Session 2	Beta-low	14	35.39 ± 32.98	50.57 ± 94.36	15.17 [-32.84, 63.19]	.507	1.000	0.18
Session 2	Beta-mid	14	34.36 ± 26.37	37.31 ± 53.43	2.95 [-27.36, 33.26]	.837	1.000	0.06
Session 2	Beta-high	14	66.43 ± 40.86	61.73 ± 65.60	-4.70 [-45.33, 35.92]	.807	1.000	-0.07
Session 2	Gamma	14	610.42 ± 1072.14	374.15 ± 291.84	-236.27 [-787.45, 314.90]	.371	1.000	-0.25

Session	Device-derived output	n	Pre, M ± SD	Post, M ± SD	Post − Pre [95% CI]	Nominal p	Holm p	dz
Session 2	Thinking index	14	68.82 ± 44.27	83.22 ± 70.17	14.40 [-14.75, 43.56]	.305	1.000	0.29
Session 2	Concentration index	14	209.80 ± 254.29	628.04 ± 1247.24	418.23 [-243.04, 1079.51]	.195	1.000	0.37

Note. Holm adjustment was applied across the 11 device-derived outputs within each session. Sub-band definitions and signal units could not be independently verified from retained manufacturer documentation. Thinking and Concentration are proprietary indices.

Table S11. Exploratory EEG log1p and Wilcoxon sensitivity analyses.

Session	Device-derived output	n	Mean log1p change	log1p p	log1p Holm p	Wilcoxon p	Wilcoxon Holm p	Raw-change Shapiro p
Session 1	Delta	19	0.377	.094	.603	.096	.669	<.001
Session 1	Theta	19	0.297	.057	.603	.134	.680	<.001
Session 1	Alpha-low	19	0.309	.066	.603	.113	.680	.001
Session 1	Alpha-mid	19	0.278	.055	.603	.032	.323	<.001
Session 1	Alpha-high	19	0.227	.068	.603	.080	.639	.071
Session 1	Beta-low	19	0.235	.068	.603	.023	.253	.026
Session 1	Beta-mid	19	0.052	.650	1.000	.984	1.000	.004
Session 1	Beta-high	19	-0.098	.355	1.000	.312	1.000	<.001
Session 1	Gamma	19	-0.071	.538	1.000	.984	1.000	<.001
Session 1	Thinking index	19	0.237	.108	.603	.055	.491	.001
Session 1	Concentration index	19	0.028	.858	1.000	.332	1.000	<.001
Session 2	Delta	14	-0.030	.909	1.000	.952	1.000	.007
Session 2	Theta	14	0.133	.594	1.000	.952	1.000	<.001
Session 2	Alpha-low	14	0.095	.619	1.000	.952	1.000	<.001
Session 2	Alpha-mid	14	0.135	.469	1.000	.761	1.000	<.001
Session 2	Alpha-high	14	0.048	.802	1.000	.952	1.000	<.001
Session 2	Beta-low	14	0.021	.910	1.000	.808	1.000	<.001
Session 2	Beta-mid	14	-0.174	.377	1.000	.268	1.000	<.001
Session 2	Beta-high	14	-0.276	.226	1.000	.268	1.000	.005
Session 2	Gamma	14	-0.175	.405	1.000	.583	1.000	<.001
Session 2	Thinking index	14	0.098	.550	1.000	.715	1.000	<.001
Session 2	Concentration index	14	0.096	.743	1.000	.952	1.000	<.001

Note. No EEG result remained significant after within-session Holm correction under the raw, log1p, or Wilcoxon analysis. These checks do not overcome single-channel acquisition or undocumented proprietary processing.

Reproducibility record.

Item	Record
Authoritative statistical source	master_data.xlsx; questionnaire, HRV, and EEG worksheets
Horticultural well-being scoring	Seven item columns; all seven required; unreadable "?" values treated as missing, not zero
Software	Python 3.12.13; pandas 2.2.3; NumPy 2.3.5; SciPy 1.17.0
Missing data	Available paired observations per contrast; no imputation
Multiplicity	One four-test Holm family for the principal DASS-21/STAI-S within-session tests; for the Session 2 three-time-point analyses, one separate three-contrast Holm family within each outcome; separate within-session Holm families for HRV and EEG.
Equivalence	Post hoc change-score TOST with illustrative dz = ±0.50 and dz = ±0.30/±0.20 sensitivity bounds. Planning values were recalculated using exact finite-sample, unknown-variance paired-difference t-TOST power under a true dz = 0, α = .05 for each one-sided test, and joint power ≥ .80: n = 36 (power = .805), 97 (.803), and 216 (.801), respectively.
Preregistration	None; outcome hierarchy and equivalence analyses were specified during revision

The anonymized data supporting the findings of this study, together with a data dictionary and analysis code, are available from the corresponding author upon reasonable request, subject to the conditions of participant consent and ethics approval.
